# An Organic Mixed Ion–Electron Conductor for Power Electronics

**DOI:** 10.1002/advs.201500305

**Published:** 2015-12-02

**Authors:** Abdellah Malti, Jesper Edberg, Hjalmar Granberg, Zia Ullah Khan, Jens W. Andreasen, Xianjie Liu, Dan Zhao, Hao Zhang, Yulong Yao, Joseph W. Brill, Isak Engquist, Mats Fahlman, Lars Wågberg, Xavier Crispin, Magnus Berggren

**Affiliations:** ^1^ Laboratory of Organic ElectronicsDepartment of Science and TechnologyLinköping UniversitySE‐601 74NorrköpingSweden; ^2^ Innventia ABBox 5604, SE‐114 86StockholmSweden; ^3^ Department of Energy Conversion and StorageTechnical University of DenmarkDK‐4000RoskildeDenmark; ^4^ Department of PhysicsChemistry and BiologyLinköping UniversitySE‐581 83LinköpingSweden; ^5^ Department of Physics and AstronomyUniversity of KentuckyLexingtonKY40506‐0055USA; ^6^ KTH Royal Institute of TechnologySchool of Chemical Science and Engineering (CHE)Fibre and Polymer Technology and Wallenberg Wood Science CenterSE‐100 44StockholmSweden

**Keywords:** conducting polymer, nanofibrillated cellulose, PEDOT, supercapacitor, transconductance

## Abstract

**A mixed ionic–electronic conductor based on nanofibrillated cellulose** composited with poly(3,4‐ethylene‐dioxythio­phene):­poly(styrene‐sulfonate) along with high boiling point solvents is demonstrated in bulky electrochemical devices. The high electronic and ionic conductivities of the resulting nanopaper are exploited in devices which exhibit record values for the charge storage capacitance (1F) in supercapacitors and transconductance (1S) in electrochemical transistors.

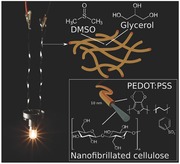

In the coming decades, a large amount of extra electrical power must be produced to cover the increasing energy requirements of our society. Various intermittent energy sources are used to produce electricity. However, because they do not fit the pattern of human activity, there is an urgent need for materials capable of storing and manipulating huge amounts of electrical energy. Electrical storage could take place in large volume electrochemical cells (batteries or supercapacitors) whose discharges are controlled through high power transistor circuits. One limitation today is identified as the absence of bulk materials with both a high electronic and ionic conduction, i.e., mixed ionic‐electronic conductor (MIEC) bulk systems. These MIECs would preferably be based on sustainable, light‐weight, and abundant materials that can be easily processed into large (even giant) volumes. Such a “green” MIEC would enable the mass adoption of supercapacitors, and may be further functionalized with catalysts for fuel cells^1^ or with additional redox species for batteries.^2^ Furthermore, this development may also help organic electronics venture into the domain of high power electronics and ultra‐low noise bioelectronic sensors.^3^

The state‐of‐the‐art in electronic, ionic and mixed conductors is summarized in **Figure** **1**. Putting aside the standard electronic and ionic conductors, MIECs belong to two distinct families: ceramics and conducting polymers. There exists a clear trade‐off between the ionic and electronic conductivities, with an unoccupied niche in the upper right corner of the graph. Ceramic materials with high ionic conductivity (points “l” in Figure 1)^4^ have been reported but are far from reaching the electronic conductivities of the best organic conducting polymers (point “o”),^5^, ^6^ although the ionic conductivity of the latter is two orders of magnitude lower. The low temperature processability (relative to ceramics) and the ease with which their wet synthesis may be scaled up makes conducting polymers attractive for mass production and implementation into giant scales.^7^

**Figure 1:**
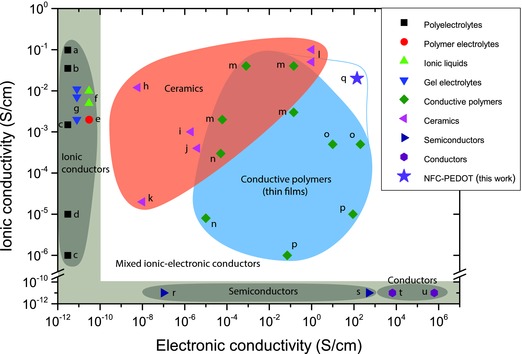
Survey of ionic and/or electronic conductors. With the exception of ionic liquids, only solid conductors are included. The points in the graph represents the following materials: a: Nafion;^20^ b: poly(diallyldimethyl ammonium chloride)/poly(2,6‐dimethyl1,4‐phenylene oxide);^20^ c: poly(4‐styrenesulfonic acid);^19^ d: poly(ethylene oxide)/poly(acrylic) acid/poly(ethylene oxide)/(poly(acrylic) acid/multiwalled carbon nanotubes);^21^ e: polyvinylidene fluoride/polyethylene oxid/propylene carbonate/LiClO_4_;^22^ f: (lithium bis(oxlate)borate and lithium tetrafluoroborate)/1‐ethyl‐3‐methyl‐imidazolium tetrafluoroborate;^23^ g: LiCF_3_SO_3_/poly(methyl methacrylate), LiClO_4_/poly(methyl methacrylate), and LiClO_4_/propylene carbonate/ethylene carbonate/dimethylformamide/poly(acrylonitrile);^24^ h: Li_10_GeP_2__2_S_12_;^25^ i: Ag_2_HfS_3_;^26^ j: Ag_2_S;^27^ k: Li_3.5_V_0.5_Ge_0.__5_O_4_;^28^ l: Ce_0.8_Gd_0.2_O_2‐d_–CoFe_2_O_4_;^1^ m: poly(3,4‐ethylenedioxythiophe­ne):polystyrene sulfonate and poly(3,4‐ethylenedioxythiophene):polystyrene sulfonate/sodium polystyrene sulfonate;^18^ n: poly‐[1‐methyl‐3‐(pyrrol‐l‐ylmethyl)pyridinium perchlorate];^29^ o: Polyaniline^2^, ^3^ p: Polypyrrole;^30^, ^31^ q: poly(3,4‐ethylenedioxythiophene):polystyrene sulfonate/nanofibrillated cellulose/dimethyl sulfoxide/polyethylene glycol (this work); r/s: GaAs;^32^ t: Nichrome;^33^ u: Ag.^33^

The development of conducting polymers, such as trans‐polyacetylene,^8^ was mainly focused on reaching high and air‐stable electronic conductivity^9^, ^10^ in more or less bulky samples.^11^ High electronic conductivity (1000 to 4000 S cm^−1^ 12, 13 has been achieved in organic thin films (10 nm to 10 μm) and in functional fibers and fibrils.^14^, ^15^, ^16^ To the best of our knowledge, there are no reports of thicker films and bulky geometries (10 μm to 10 cm). The methods by which thin‐films are fabricated are ill‐suited to produce thick films mainly because they would rely on a multistep process. Such multilayer films would also suffer from internal mechanical stresses that lead to delamination and cracking. Organic electronics currently focuses on ultrathin transparent electrodes for the replacement of expensive transparent metal oxide electrodes in solar cells and light‐emitting diodes.

In parallel to these developments, (semi)conducting polymers have been investigated for their reversible electrochemical activity due to the fact that they are intrinsic MIECs. One strategy to improve the ionic conductivity and the aqueous processability has been to composite a polyelectrolyte with a conjugated polymer.^17^ Poly(3,4‐ethylene‐ dioxythiophene):poly(styrene‐sulfonate) (PEDOT:PSS) is the most studied and used conducting polymer (point “m”).^18^ In those blends, the electronic conductivity is strongly correlated with the phase separation. The latter can be controlled and suppressed with the addition of high boiling point solvents. This effect is called “secondary doping” to distinguish it from the “primary doping” which controls the charge carrier density in the PEDOT phase. Importantly, despite the addition of PSSH in those blends, the ionic conductivity is limited to 1.5 mS cm^−1^ at 80%RH^19^ which is still well below the values reported for ceramics (see Figure 1).

In this work, we present a composite system, comprised of PEDOT:PSS, nanofibrillated cellulose (NFC), glycerol, and dimethylsulfoxide (DMSO) blended from solutions. This malleable NFC‐PEDOT:PSS‐glycerol‐DMSO composite (hereafter termed NFC‐PEDOT paper for brevity), combines high electronic and ionic conductivity with facile manufacturing, mechanical properties compatible with paper‐making machines, and ease‐of‐handling. In NFC‐PEDOT paper, PSS acts as a polyanionic counterion to neutralize the highly oxidized PEDOT chains.^34^ DMSO is a high‐boiling point solvent commonly used as a conductivity enhancement agent for PEDOT:PSS.^35^ This secondary dopant impacts the nano‐ and micromorphology of PEDOT:PSS by improving crystallinity (π‐stacking) and promoting phase separation of the PSS in excess to promote the creation of highly conductive percolation paths. NFC is a nanofiber scaffold system with “remarkably high toughness with a large strain‐to‐failure.”^36^ NFC is here utilized as a 3D‐scaffold to improve the PEDOT:PSS's micro/mesoscopic organization in 3D (i.e., it acts as a tertiary dopant to favor high conductivity also in bulky dimensions). Adding glycerol improves the composite's plasticity while increasing its hygroscopicity, which allows ions to move much easier.

The MIEC composite is made from a PEDOT:PSS aqueous solution (1.3 wt%, Clevios PH 1000) and an aqueous solution of 0.1 wt% nanofibrillated cellulose NFC. The anionic charge of the NFC is around 600 μeq. g^−1^. The PEDOT:PSS and NFC solutions are mixed together with glycerol and dimethylsulfoxide (DMSO) in the following weight percentages: 13.2/6.1/9.5/68.2 (wt%). The solution is homogenized with a high‐shear batch mixer and cast into a petri dish. After the solvent has evaporated at room temperature and atmospheric pressure for 48 h, a self‐supporting film is obtained with a weight percent ratio of 19.2/7.3/73.5 for PEDOT:PSS/NFC/(DMSO+glycerol) (see Figure S1, Supporting Information). Because solution‐casting is a scalable method, it is possible to produce films with a wide range of thicknesses and geometries. **Figure** **2**a shows a cross‐section of three samples of different thicknesses (60, 250, 8500 μm) made by varying the volume of the cast solution. The resulting NFC‐PEDOT is a material that combines the advantageous properties of cellulose and conducting polymers. The NFC‐PEDOT paper may be folded (even creased) repeatedly while retaining its mechanical and electrical properties. Figure 2b shows NFC‐PEDOT folded into an origami swan, a treatment which is impossible to apply to a thick PEDOT:PSS film because of its brittleness. The NFC‐PEDOT can be used as an electrical conductor which supplies current to an (opto)electronic device (Figure 2c). The NFC‐PEDOT composite is mechanically resilient (Figure 2d,e) and exhibits the following properties: a Young's modulus of *E* = 0.66 ± 0.05 GPa, a tensile strength of σ_T_ = 13.5 ± 1.7 MPa, and strain at break of ε_T_ = 13.4% ± 2.0% (the stress‐strain curves from which these values were derived can be found in Figure S2, Supporting Information). The NFC‐PEDOT paper is slightly weaker compared to typical copy paper but its mechanical properties are sufficient for practical handling and processing in a paper machine. Finally, the composite's cohesiveness does not degrade in water (see Figure S14, Supporting Information). This structural integrity is crucial for the implementation of bulk electrochemical devices. We explain the unique combination of mechanical and MIEC properties through a material model sketched in Figure 2f in which the conducting polymer PEDOT:PSS forms a homogeneous coating around entangled cellulose nanofribrils.

**Figure 2:**
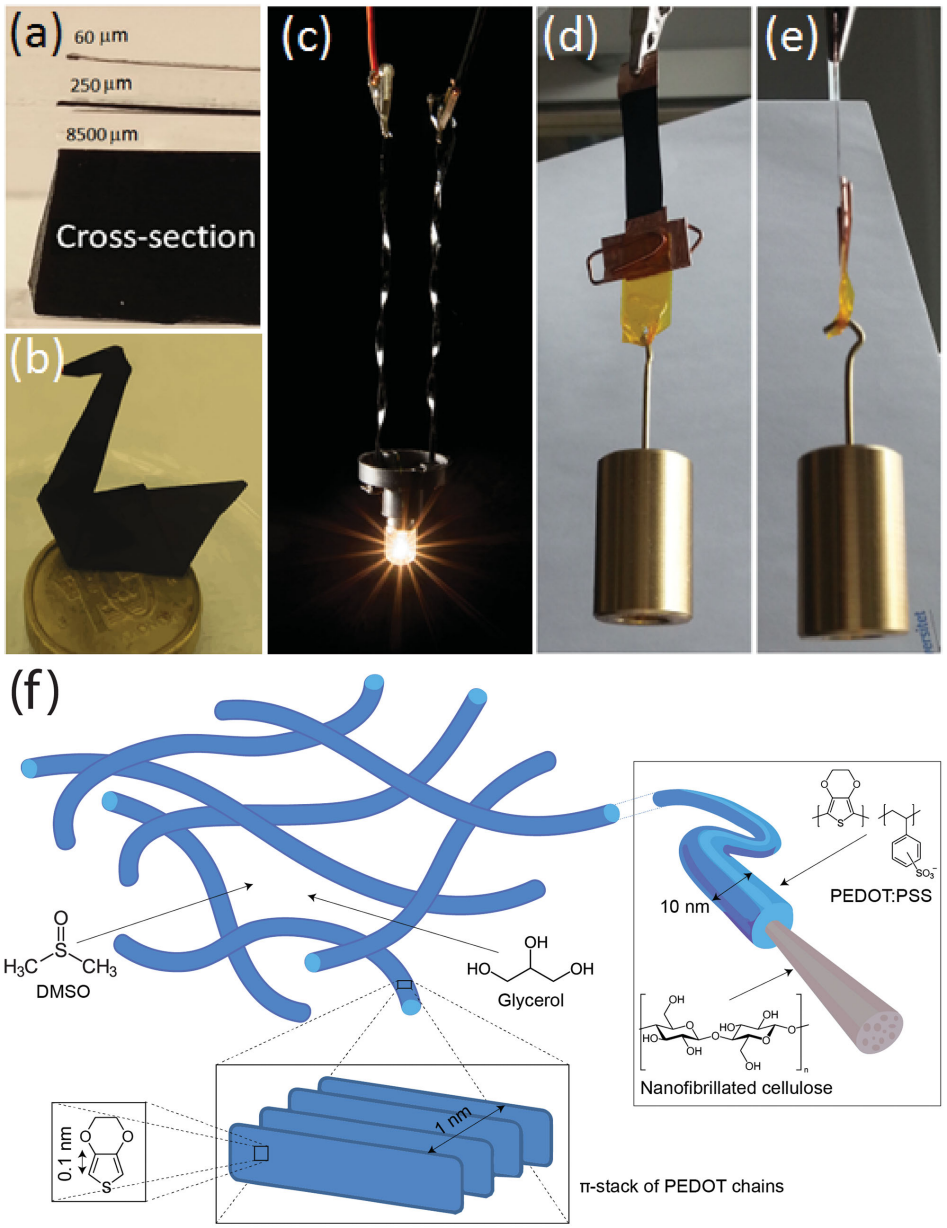
a) Cross section showing a wide range of thicknesses for the NFC‐PEDOT nanocomposite, b) NFC‐PEDOT origami structure, c) NFC‐PEDOT ribbons connected to a LED, d,e) 122 μm thick NFC‐PEDOT stripe subjected to a weight of 100 g. f) Proposed multiscale model for the morphology and self‐organization in the NFC‐PEDOT composite. The chemical structures of each of the component are indicated.

The model in Figure 2f is supported by the results of a thorough microscopic and spectroscopic investigation. First, atomic force microscopy (AFM) shows the presence of individual cellulose nanofibrils (10–20 nm diameter) forming an entangled network (**Figure** **3**a). NFC samples without the PEDOT:PSS phase and without high boiling point solvents, in contrast, display densely packed nanofibers (Figure S3, Supporting Information). These observations suggest that the PEDOT:PSS coaxial coating and the presence of DMSO/glycerol weaken the interaction between the cellulose nanofibers (i.e., prevents the nanofibers from aggregating). Figure 3b shows results from an elemental analysis of the NFC‐PEDOT surface using X‐ray photoelectron spectroscopy (XPS). The C(1s), O(1s), S(2p) core level spectra reveal the elements' chemical fingerprint.^37^ The S(2p) signal is solely attributable to the PEDOT:PSS with two contributions (doublets): (i) the sulfur atom in the thiophene ring of PEDOT (164 eV) or (ii) the sulfonate group of PSS (168 eV). The comparison between these spectra indicates that the outer surface (within a depth of 5–10 nm) of the NFC‐PEDOT composite consists of PEDOT:PSS only. The XPS peak integrated intensities give the atomic ratios C/O/S of the various samples. For the NFC‐PEDOT paper, these were found to be 61.3/28.8/9.9, which is very similar to the ratios obtained from a pure PEDOT:PSS layer (61.5/28.2/10.3). These ratios, however, are clearly different from a pure NFC layer deposited on Au (54.9/44.7/0.35). The wide scan XPS data are shown in the Supporting Information (Figure S4, Supporting Information). Finally, UV‐photoelectron spectroscopy (Figure S5, Supporting Information) indicates that the valence band of the composite has no band gap since a density of states is detected at the Fermi level of the spectrometer. This is a previously reported feature of conducting materials such as PEDOT:PSS.^9^ Therefore, XPS/UPS analysis further confirms that a PEDOT:PSS layer is fully coating the cellulose nanofibrils. We hypothesize that PEDOT:PSS has self‐organized to form a continuous cladding layer around the NFC fibers during the preparation of the material, i.e., upon mixing the two components in an aqueous suspension. Wide angle X‐ray scattering in transmission (WAXS) and at Grazing Incidence (GIWAXS) were used to characterize the crystallinity and texture of the NFC‐PEDOT paper. The self‐supporting films containing NFC clearly indicate a uniaxial orientation with a sharp, well‐defined peak corresponding to the cellulose II *004* reflection.^38^ This peak is only observed in the transmission measurement, and with the *110* and *020* reflections preferentially oriented along the surface normal, as clearly seen in the GIWAXS measurement (Figure 3d). This indicates strong texturing, with long fiber dimension of the NFC oriented parallel with the film surface. The PEDOT:PSS shows a clear π‐stacking peak at a scattering vector length (Q) of 1.8 Å^−1^ (Q = 4πsinθ/λ, where 2θ is the scattering angle, and λ the X‐ray wavelength of 1.5418 Å). The PEDOT:PSS reflection visible in both WAXS and GIWAXS measurements shows very little tendency of preferred orientation and indicates that the crystalline PEDOT domains are randomly oriented. This is consistent with the observation that PEDOT:PSS fully covers the NFC fibers. WAXS and GIWAXS data for the various combinations of NFC, PEDOT:PSS and glycerol in film were acquired to assist in the identification of the crystalline components (not shown).

**Figure 3:**
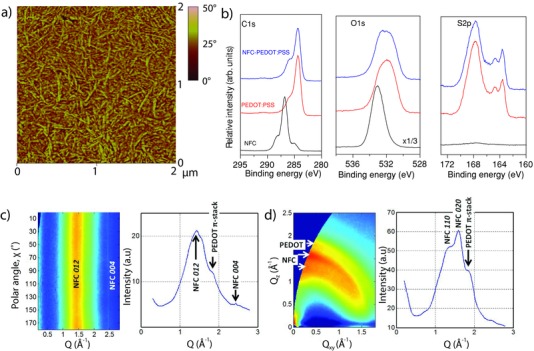
a) Phase AFM image of a 70 μm thick film of vacuum‐dried NFC‐PEDOT composite (topography image is in Figure S3, Supporting Information); b) XPS(AlKα) C(1s), O(1s), and S(2p) core‐level spectra for three different films: NFC‐PEDOT, PEDOT:PSS and NFC; c) WAXS data taken in transmission and integrated along 180° along the powder rings (left) with sum of all azimuthal bins (right); and d) 2D GIWAXS data (left) and corresponding azimuthal integration ±30° with respect to the surface normal (right).

High electronic conductivities in thin films typically originate from thin‐film processing protocols that use the substrate to promote self‐organization,^39^ as well as a phase separation which promotes a strong anisotropy in conductivity.^40^ For thick films, one expects to partially lose the template effect of the substrate upon coating.^41^ We investigate whether cellulose nanofibers may act as a nanoscale 3D substrate for PEDOT:PSS to promote orientation and ordering of the conducting polymer which would, in turn, favor high conductivity. **Figure** **4**a shows the temperature dependence of the electrical conductivity for a 5 μm thick layer of NFC‐PEDOT after drying the sample in vacuum (50 °C for 72 h) to remove the solvent. The conductivity σ is high (420 S cm^−1^ at 20 °C) and, at low temperatures, exhibits the characteristics of metallic‐type charge transport (σ decreases as T increases). This metallic transport at low temperature has been observed for PEDOT derivatives of higher crystallinity than that found in PEDOT:PSS.^9^ The fact that we observe metallic conductivity strongly indicates that the NFC acts as a template which promotes self‐organization of the PEDOT chains. We label the NFC a *tertiary dopant* for PEDOT, where PSS and DMSO are the primary and secondary ones, respectively.^42^ From the composition of the annealed composite (57.5 wt% PEDOT:PSS), we calculate the effective conductivity of PEDOT:PSS as σ_eff_ = σ/0.575 = 730 S cm^−1^. This value is the same as the one obtained when adding DMSO to Clevios PH 1000 (in the same ratio as NFC‐PEDOT), and not far from optimized commercial PEDOT:PSS (850 S cm^−1^). We hypothesize on the mechanism behind the tertiary doping effect as follows: Being negatively charged with carboxylic groups (degree of substitution DS = 0.1), NFC fibers may partially replace PSS as the primary dopant. Furthermore, the positively charged PEDOT chains may self‐organize along the NFC fibers.

**Figure 4:**
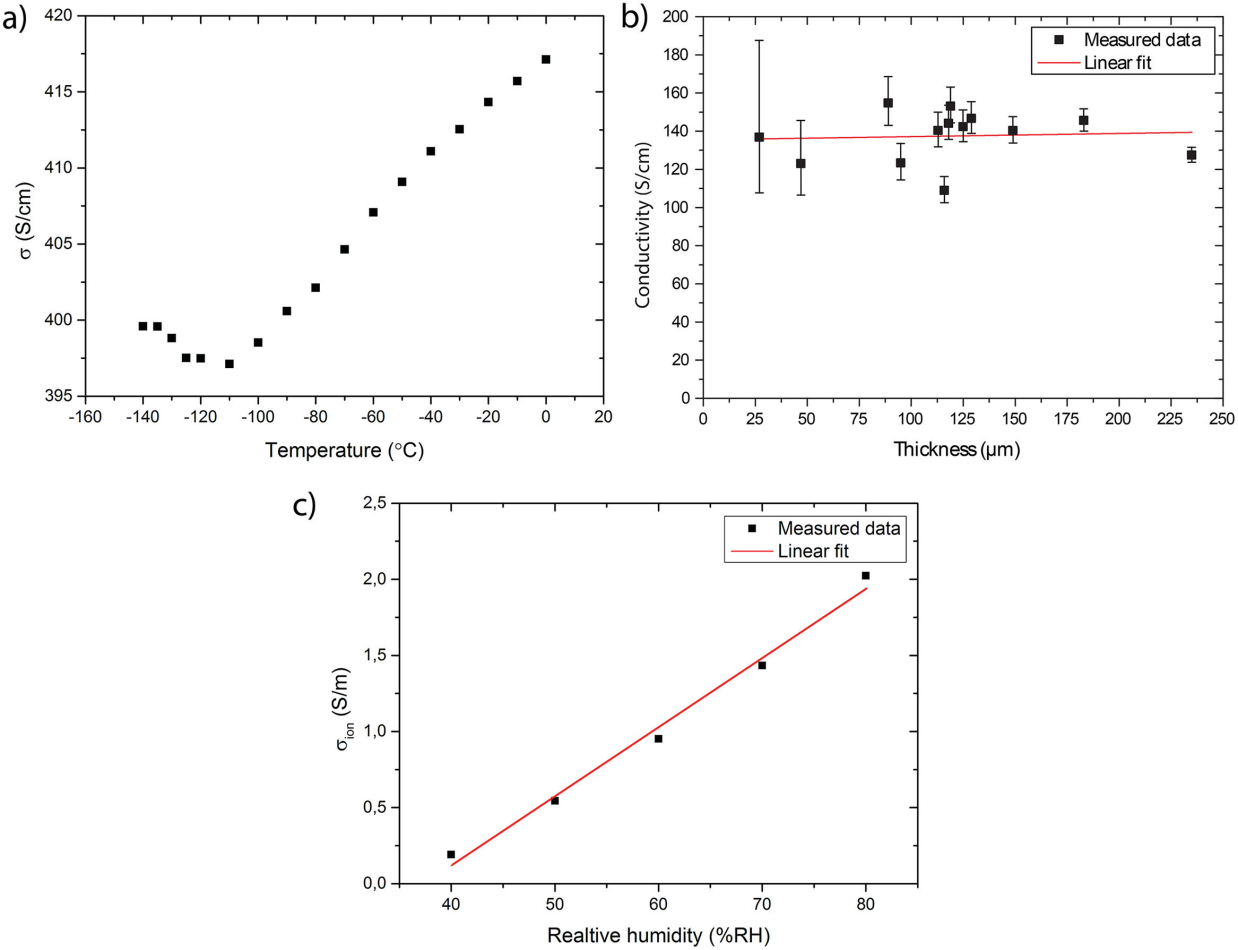
a) Electrical conductivity versus temperature for a 5 μm layer of NFC‐PEDOT dried in vacuum. b) Conductivity versus thickness for the NFC‐PEDOT composite at 40%RH and 22 °C. c) Ionic conductivity versus RH (at 1 kHz) as characterized by impedance spectroscopy for a NFC:PSSH composite (i.e., sans PEDOT).

Removing solvents from the composite makes it firmer and is expected to limit the ionic transport, which is not desirable for electrochemical devices. For that reason, we focus on NFC‐PEDOT paper which has not been vacuum dried. Conductivity measurements were performed for several sample thicknesses (>20 μm). The results (Figure 4b) show that the overall conductivity is fairly constant over the whole thickness range examined (137 S cm^−1^). The systematic error shown in Figure 4b derives from the film thickness measurement (see the Supporting Information text for details). The parameters of the regression analysis are provided in Table S2 of the Supporting Information. The lower electronic conductivity compared to the vacuum dried samples may be explained by the presence of solvents (DMSO, glycerol) in the interstitial space between the conducting nanofibers which leads to less points of contact between the chains of PEDOT. The temperature dependence on electrical conductivity σ for a 70 μm thick NFC‐PEDOT layer follows an Arrhenius behavior (log σ is proportional to 1/T) specific to the nearest neighbor hopping transport regime^40^ (activation energy of 3.75 meV, see Figure S6, Supporting Information). Electronic transport between PEDOT:PSS‐coated NFC fibers is most likely the limiting factor. The role of DMSO and glycerol is expected to be the removal of excess PSS from the PEDOT:PSS,^43^ resulting a PSS‐rich phase between the coated NFC fibers.

The presence of high boiling point solvents and the excess of PSS located in the interstitial space are expected to lead to a high ionic conductivity of the NFC‐PEDOT paper. In order to estimate the ionic conductivity, we made a NFC‐PSSH paper with the same composition as NFC‐PEDOT paper but without the PEDOT phase. The NFC‐PSSH film shows similar fibrillary structure as films of NFC‐PEDOT (see Figure S3e,f, Supporting Information). The diameter and separation of the fibers in NFC‐PSSH and NFC‐PEDOT (Figure S3b,f, Supporting Information) are similar. The solvent‐filled interstitial volume between the fibers, where most of the ionic transport occurs, is of the same order of magnitude in NFC‐PEDOT and NFC‐PSSH. Figure 4c shows the ionic conductivity of the resulting NFC‐PSSH paper versus RH as characterized by impedance spectroscopy. The ionic conductivity increases linearly from 2 mS cm^−1^ at 40%RH up to 20 mS cm^−1^ at 80%RH. For comparison, the conductivity of seawater (0.5 m NaCl) is around 50 mS cm^−1^, while that of a PSS:H thin film varies exponentially from 0.7 mS cm^−1^ at 40%RH to 1.5 mS cm^−1^ at 80%RH.^19^ NFC‐PSSH has one order of magnitude higher ionic conductivity compared to PSSH. We attribute this enhancement to the presence of interconnected solvent‐filled nanovoids forming a channel system for fast ion transport. The voids originate from the interstitial space between the cellulose nanofibers. Although NFC‐PSSH and NFC‐PEDOT papers included a nanoscopic liquid phase, they are paper‐like to the touch (i.e., not wet). As indicated by the blue star in the upper right corner of Figure 1, this material combines high electronic and ionic conductivity that reaches 140 S cm^−1^ and 20 mS cm^−1^, respectively. This material may be classified as a superionic conductor.^25^

The NFC‐PEDOT paper was used as resistor. In Figure S8 of the Supporting Information, we show an IR‐camera image of a 160 μm thick stripe carrying 1 A. Compared to most inorganic resistors, this NFC‐PEDOT one has a very low thermal conductivity (11.6 ± 1.0 mW cm^−1^ K^−1^, see Figure S7, Supporting Information), which leads to an extreme Joule effect (Figure S8a,b, Supporting Information). In the following, we summarize various key thermal and thermoelectric properties for this composite: specific heat *c* = (1.32 ± 0.4) J g^−1^ K^−1^ at 298 K, in‐plane thermal diffusivity, *D* = (7.0 ± 0.2) × 10^−3^ cm^2^ s^−1^, mass density *ρ* = (1.26 ± 0.4) g cm^−3^, and the Seebeck coefficient is ≈17 μV K^−1^.

The use of PEDOT:PSS as charge storage material in a supercapacitor has been previously reported.^44^ When the charge concentration in PEDOT is reduced or increased, compensating ions will move in and out of the material resulting in a pseudocapacitive (faradaic) charge storage reaction. The NFC‐PEDOT paper with enhanced ionic conductivity and mechanical properties compared to PEDOT:PSS allows scaling up thin film supercapacitor into bulky supercapacitor electrodes. **Figure** **5**a depicts a schematic of a supercapacitor cell (and three‐electrode electrochemical setup) used to characterize the electrochemical properties of the nanopaper. Figure 5b shows the charging and discharging curves of a supercapacitor made from electrodes of NFC‐PEDOT (≈71 cm^2^). A photograph of the actual device and its electrodes is shown in Figure S11 of the Supporting Information. The device may store up to 1.2 C of charge with a capacitance of 2 F. This is the highest reported value of charge stored in a supercapacitor made from conjugated polymers. The material displays highly capacitive behavior in the 0–0.6 V potential range as indicated by a square shaped voltammogram (see inset of Figure 5c). Figure 5c shows a linear dependence of the capacitance with the thickness of the NFC‐PEDOT paper electrodes (of area ≈1 cm^2^). This linearity indicates that the electrochemical reaction occurs throughout the entire bulk of the electrodes. Figure 5d displays the stored and released charge as well as the charge retention during chronoamperometric cycling and shows excellent charge retention (>99% over 500 cycles). The water stability of the composite is qualitatively superior to that of untreated PEDOT:PSS (Figure S14, Supporting Information). The excellent stability of the NFC‐PEDOT capacitors in aqueous electrolytes is attributed to the strong binding effect of the NFC fibrils on the PEDOT chains and domains as previously observed for cellulose composites.^45^ The flexibility of this composite immunizes it against the structural‐change‐driven degradation that plagues charge storage media.^46^ The electrical and electrochemical behavior of the supercapacitors was modeled by equivalent circuits using the chronoamperometric charging measurements (Figure S12, Supporting Information) and impedance spectroscopy measurements (Figure S13, Supporting Information). The time constant and the theoretical minimum time constant of these models are calculated as 400 and 40 ms, respectively.

**Figure 5:**
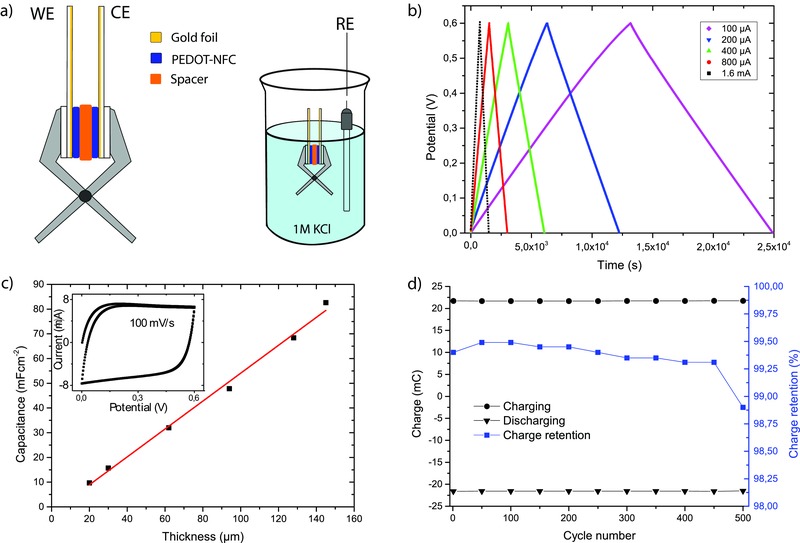
a) Supercapacitor cell and three‐electrode‐electrochemical setup, b) galvanostatic charge–discharge measurements on large capacitor, c) capacitance versus electrode thickness (inset: voltammogram for a 130 μm thick film), and d) cycling stability measurements.

Organic electrochemical transistors (OECTs) are a fundamental building block for printed sensors and logic circuitry. The channel of the OECTs is a mixed conductor whose electronic transport is modulated by charging a capacitor through the gate voltage. OECTs based on PEDOT:PSS are p‐type devices that operate in depletion mode. Because the charge carrier concentration is homogeneously modulated throughout the entire bulk of the channel, OECTs based on PEDOT:PSS have been shown to exhibit the largest transconductance amongst all transistor technologies.^47^, ^48^ These high‐gain devices have been touted as transducers in biosensing applications, since OECTs outperform both well established as well as emerging technologies in this regard.^47^

Transconductance is defined as the ratio of the modulation in output current to the input's change in potential (see Equation (1)). Equation (2) relates the transconductance to different device parameters (*w* width, *t* thickness, *L* length, *ρ_1_* resistivity of reduced state, *ρ*_2_ resistivity of oxidized state)

(1) $$g_{m}\;=\;\frac{\partial I_{\mathrm{out}}}{\partial V_{\mathrm{in}}}$$

(2) $$g_{m}\propto \frac{\rho_{1}-\rho_{2}}{\rho_{1}\rho_{2}}\frac{wt}{L}$$

The scalability and high current density of the NFC‐PEDOT composite allows us to fabricate an OECT with a giant transconductance in excess of 1 S, with the device structure shown in the inset of **Figure** **6**a. This was achieved by using device dimensions of *w* = 55 mm, *t* = 200 μm, *L* = 1 mm, and maximizing the on‐current (≈1 A). We used an aqueous polyelectrolyte (10 wt% [Poly(vinylpyrrolidone‐co‐N‐methyl‐vinylimidazolium)]n+ n[chloride]− in water) and NFC‐PEDOT as both the channel and gate material.

**Figure 6:**
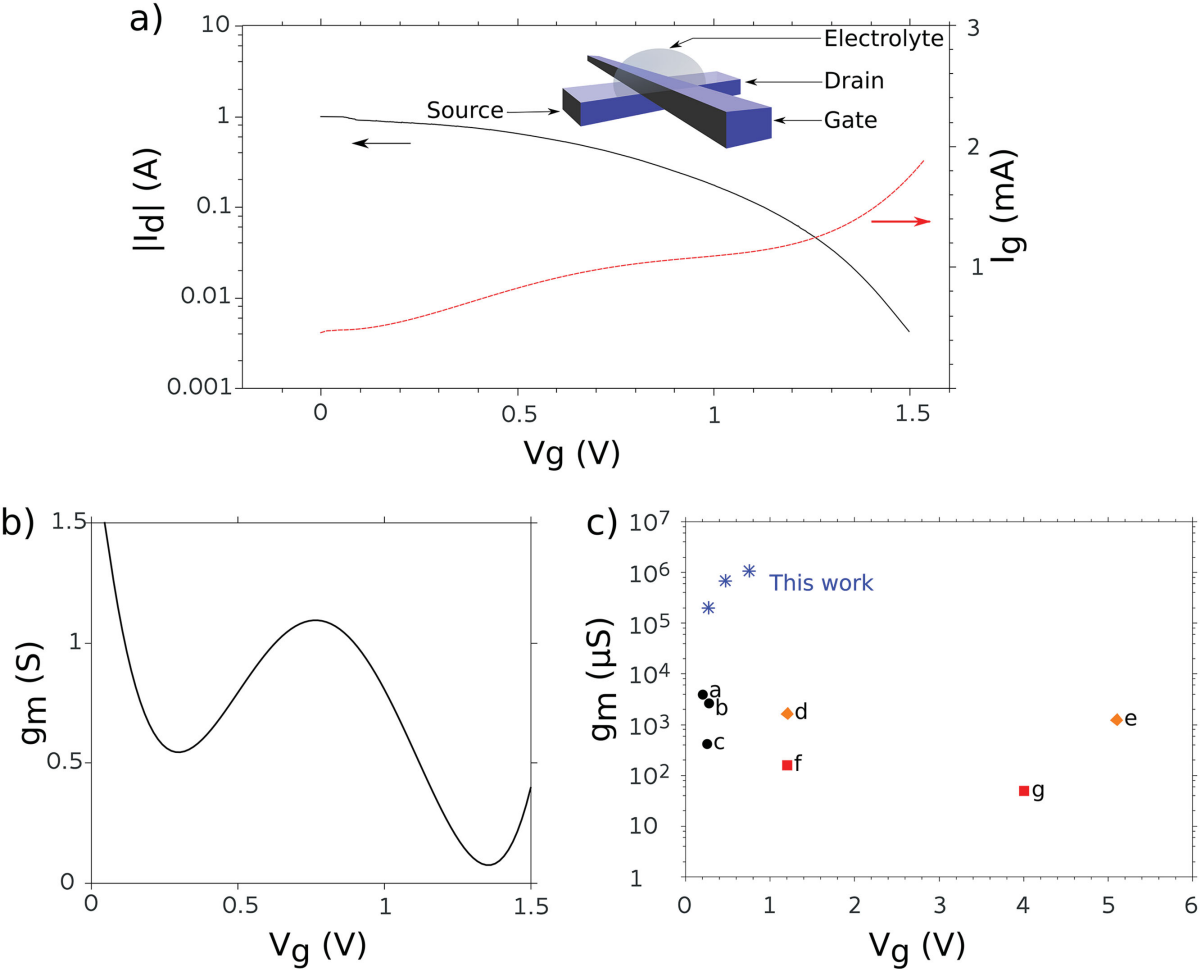
Giant transconductance organic electrochemical transistor. a) Transfer curve of the transistor. b) Transconductance. c) Survey of the highest reported transconductance values (versus gate voltage). Blue asterisks represent this work, while other technologies represent the following semiconductor/dielectric couple: a: PEDOT:PSS/Saline (best);^47^ b: PEDOT:PSS/saline (typical);^47^ c: Graphene/PBS + NaCl;^49^ d: Graphene/SiO_2_;^50^ e: ZnO/Al_2_O_3_;^51^ f: ZnO/DEME‐TFSI;^52^ g: P3HT/EMIM‐TFSI.^53^

Figure 6a shows the transfer curve (with gate current) and Figure 6b shows the transconductance curve clearly exhibiting a value of >1 S at ≈0.8 V. Figure 5c surveys the transconductance versus gate voltage of different state‐of‐the‐art transistor technologies, where the black circles represent transistors that use aqueous electrolytes as dielectrics, the transistors depicted with orange rhombi use oxides, while the red squares show the ones with ionic liquids.^47^ The blue asterisks, reaching two orders of magnitude higher transconductance than the state‐of‐the‐art represent three different versions of the transistor shown in Figure 6a,b.

In summary, we developed a scalable, “true bulk,” flexible yet robust mixed ionic‐electronic conductor paper with an outstanding combination of electronic and ionic conductivities. This enables organic and paper electronics to transcend the domain of thin films and move into the third dimension, a crucial step for mass storage applications and enabling power electronics with organic materials. The ratio between the electronic‐to‐ionic conductivity could be optimized as electrodes or semiconductors for various applications ranging from fuel‐cells, transistors and sensors, supercapacitors to batteries.

## Supporting information

As a service to our authors and readers, this journal provides supporting information supplied by the authors. Such materials are peer reviewed and may be re‐organized for online delivery, but are not copy‐edited or typeset. Technical support issues arising from supporting information (other than missing files) should be addressed to the authors.

Supplementary
