# Supplementary material for: An Organic Mixed Ion–Electron Conductor for Power Electronics
Source: Adv Sci (Weinh). 2015 Dec 2;3(2):1500305. doi: 10.1002/advs.201500305 (PMC5063141; doi:10.1002/advs.201500305)
Supplement: Supplementary file 1 — Supplementary [file ADVS-3-0g-s001.pdf]

Copyright WILEY-VCH Verlag GmbH & Co. KGaA, 69469 Weinheim, Germany, 2015.

ADVANCED SCIENCE
Open Access

Supporting Information

for *Adv. Sci.*, DOI: 10.1002/advs. 201500305

An Organic Mixed Ion–Electron Conductor for Power Electronics

Abdellah Malti, Jesper Edberg, Hjalmar Granberg, Zia Ullah Khan, Jens W. Andreasen, Xianjie Liu, Dan Zhao, Hao Zhang, Yulong Yao, Joseph W. Brill, Isak Engquist, Mats Fahlman, Lars Wågberg, Xavier Crispin, and Magnus Berggren*

Supplementary Information for
An Organic Mixed Ion-Electron Conductor for Power Electronics

Abdellah Malti[a†], Jesper Edberg[a†], Hjalmar Granberg[c], Zia Ullah Khan[a], J. W. Andreasen[d], Xianjie Liu [b], Dan Zao[a], Hao Zhang[e], Yulong Yao[e], J.W. Brill[e], Isak Engquist[a], Mats Fahlman[b], Lars Wågberg [f], Xavier Crispin[a*], Magnus Berggren[a]

† These authors contributed equally to this work.
*Corresponding author: xavier.crispin@liu.se

[a]Linköping University
Department of Science and Technology, Organic Electronics
SE-601 74 Norrköping, Sweden
Equal contribution

[b]Linköping University
Department of Physics, Chemistry and Biology
SE-581 83 Linköping, Sweden

[c]Innventia AB, Box 5604
SE-114 86 Stockholm, Sweden

[d]Department of Energy Conversion and Storage
Technical University of Denmark
4000 Roskilde, Denmark

[e]Department of Physics and Astronomy,
University of Kentucky, Lexington,
KY 40506-0055, USA

[f]KTH Royal Institute of Technology
School of Chemical Science and Engineering (CHE),
Fibre and Polymer Technology, and Wallenberg Wood Science Center
SE-100 44 Stockholm

This file includes:

Materials and Methods
Supplementary Text
Figures S1 to S14
Tables S1 to S3

Materials and Methods

Material fabrication

PEDOT:PSS solution, Clevios PH 1000, was purchased from Heraeus . Glycerol, dimethyl sulfoxide (DMSO) and polystyrene sulfonic acid (PSSH) were purchased from Sigma-Aldrich. Nanofibrillated cellulose (NFC) was produced by a high pressure homogenization of cellulose dispersed in water at 1700 bar. The resulting concentration of the NFC dispersion was 0.1 wt%. Clevios PH 1000, NFC solution, glycerol and DMSO were mixed in a solid content (excluding only water) weight ratio of 16.2/6.1/9.5/68.2. The solution was homogenized using a T 10 basic ULTRA-TURRAX (Laboratory Mixers) for several minutes followed by degassing for 1 hour in a vacuum desiccator. The solution was then cast in plastic petri dishes (5.5 cm diameter and 30 ml volume) and left to dry for a week in a fume hood. After drying, the films were baked in an oven at 50°C for 5 minutes before peeling of the film from the petri dish.

Films with different thicknesses were manufactured by adding different volumes of solution to the petri dishes or by modifying the solid content concentration of the solution. For the thinnest films, the solution was diluted with water before mixing. For the thickest films, the NFC solution was concentrated to a concentration of 0.5 wt% before mixing. This was done by evaporating water from the solution while stirring. In doing so, the volume of the solution could be reduced to fit the 30 ml petri dishes.

A freestanding film of PEDOT:PSS was also made by pouring 4.7g of Clevios PH 1000 solution into a petri dish and leaving it to dry.

A NFC-PSSH composite was fabricated with the same proportions of PSSH, NFC, glycerol and DMSO as in the NFC-PEDOT composite using the same manufacturing technique.

Tensile testing

The conditioned (23ºC, 50% RH) 120 µm thick samples were cut into 50×6.1 mm test pieces which were mounted in the MTS tensile tester at a clamping distance of 30 mm. The setup was equipped with a 1250 N measurement cell and the pulling speed was set to 100% per minute. Data for 12 separate samples were collected to monitor statistical variations.

GIWAXS

Grazing Incidence Wide-Angle X-ray Scattering (GIWAXS) experiments were performed on a custom-built setup at DTU Energy, based on a rotating Cu-anode from Rigaku operated in point focus at 50 kV/200 mA, equipped with a 1D multilayer mirror from Xenocs yielding focused and monochromatic X-ray Cu Kα radiation (wavelength $\lambda = 1.5418$ Å). The incidence angle used was 0.18° with respect to the surface of the freestanding films, mounted between clamps without tension. Transmission measurements were performed at normal incidence. The measurements were done in vacuum, at room temperature. The scattered radiation was collected using a Fuji imaging plate placed 119 mm from the sample.

Photoelectron spectroscopy

Photoemission experiments are carried out using a Scienta® ESCA 200 spectrometer in ultrahigh vacuum with a base pressure of 1×10^{-10} mbar. The measurement chamber is equipped with a monochromatic Al K_α x-ray source providing photons with 1486.6 eV. The XPS experimental condition is set so that the full width at half maximum of the clean Au $4f_{7/2}$ line was 0.65eV. The pure NFC samples were insulating and a flood gun was used in the XPS experiments for these samples. UPS was carried out using monochromatized He I (hv=21.22 eV) photons from a He resonance lamp. The total energy resolution of the UPS measurements was set to 0.1 eV

AFM

Morphological AFM measurements were performed using a Dimension 3100 microscope equipped with a Nanoscope III controller from Bruker-Nano. All images were recorded under ambient conditions. In Intermittent Contact AFM (tapping-mode) mode, Si cantilevers with a resonance frequency in the 150-300 kHz range are used. All the measurements are done with the scan size of $2000 \times 2000 nm^2$.

Specific heat and thermal diffusivity

The specific heat, with precision 3%, was measured using differential scanning calorimetry[1] on a pellet prepared from pressing several sheets of nanopaper together, with results shown in the inset to Fig. S7. Note the transition at T ~ -50 ^{o}C presumably due to freezing of the glycerol in the matrix.

We have measured the in-plane thermal diffusivity (D) by the ac-calorimetric technique of Hatta et al. [2] The front of the sample is heated with light that is chopped at frequency f and the conducted oscillating temperature (T_{ac}) reaching the back of the sample is measured with a small thermocouple glued onto the sample with conducting paint. A movable screen blocks part of the sample. When the thermocouple is behind the screen, $d\ln V_{ac}/dx = (\pi f/D)^{1/2}$, where V_{ac} is the magnitude of the oscillating thermocouple voltage. We measured a few samples of different thicknesses with consistent results. Experimental results for a 30 μm thick sample are given in Fig. S7. For small values of x, the signal saturates as the edge of the screen overlaps the glue attaching the thermocouple, while for large x the signal becomes comparable to the noise and offset voltage of the measuring electronics.[3] The slopes in the intermediate, linear regions $f^{-1/2} d\ln V_{ac}/dx = (2.12 \pm 0.03) / mm \cdot Hz^{1/2}$ are used to determine D.

Electrical and ionic conductivity

The in-plane conductivity of the composite was measured on a Keithley 4200-SCS parameter analyzer using the four-probe resistance measurement technique. Four evenly spaced gold contacts were evaporated onto PET plastic sheets. The composite films were laminated on the contacts using a carbon paste as glue to ensure good contact between the gold and the samples. Prior to measuring, each sample was baked for 1 minute at 50°C to remove any residual water. This was done to prevent conductivity modulation during the measurements due to water evaporating from the samples.

The temperature dependence on the conductivity was investigated using a cryogenic probe station (Janis Research) together with the aforementioned parameter analyzer. Two samples were prepared; one freestanding 70 µm thick sample of NFC-PEDOT film glued

onto gold electrodes using carbon paste and one 5 µm sample that was drop-cast onto the electrodes. The temperature was scanned from 0°C to -140°C in steps of 10°C. The conductivity was measured at each step. The 5 µm sample was baked at 50°C in vacuum for 72h to ensure that all solvents had evaporated. After annealing the thickness of the film was 4 µm and the same conductivity measurement was performed again.

The main component of the error in Fig. 4b is due to thickness measurement and was assumed to be systematic. The thickness measurement error was calculated from measurements on 15 different spots on a representative sample. This error was then used to calculate the error in conductivity. The variation in thickness observed is due to sample inhomogeneity as well as from a human error when using a micro caliper on such soft material.

The in-plane ionic conductivity of the NFC-PSSH composite was measured using impedance spectroscopy. The samples were prepared in the same manner as for the electronic conductivity measurements, though only two probes were used for this measurement. The samples were kept in an ETC-04SH-JY-CE climate chamber (Blue M Electric) and the measurements were performed on an Alpha High Resolution Dielectric Analyzer (Novocontrol). The impedance spectrum was recorded while sweeping the frequency from 1 MHz to 100 mHz. This was done for a number of humidity levels in the climate chamber. The conductivity was calculated from the real part of the impedance at 1 kHz.

<u>Electrochemical cells</u>

Au was evaporated onto PET foil and was used as current collectors for the electrochemical cells. The NFC-PEDOT films were laminated onto the gold foil and two such electrodes were sandwiched together with a porous cellulose spacer in between to form a capacitor structure. The capacitors were then sandwiched between two rigid plastic plates and the whole structure was held together by a clamp. Three large circular capacitors (5.5 cm diameter) and a number of smaller square (1 cm^2) capacitors were manufactured. The smaller capacitors were made with a number of different NFC-PEDOT film thicknesses. A capacitor made with the freestanding PEDOT:PSS film was also manufactured.

<u>Electrochemical measurements and equipment</u>

All electrochemical measurements were done on a µAUTOLAB potentiostat (Metrohm) in 1M aqueous KCl solution. Galvanostatic charge-discharge measurements were made with a two electrode setup (with counter electrode and reference electrode connected). The tree large circular capacitors were connected in parallel, effectively forming a single capacitor with a surface area of 71 cm^2. The measurements were performed in the potential range 0V to 0.6V at increasing current biases. Galvanostatic charge-discharge measurements were also performed on the 1 cm^2 capacitors with different film thicknesses as well as on the capacitor with only Clevios PH 1000. Chronoamperometric charge-discharge measurements were performed with a two electrode setup on a 1 cm^2 capacitor. The capacitor was charged at 0.6V and discharged at 0V over 500 cycles.
Cyclic voltammetry was performed with a three-electrode setup with an Ag/AgCl/KCl reference electrode on a 1 cm^2 capacitor in the potential range 0V to 0.6V at a rate of 100

mV/s.
Electrochemical impedance spectroscopy was performed with a two-electrode setup on a 1 cm^2 capacitor in the frequency range 100 kHz to 100 mHz.

Infrared images:

All infrared images were acquired with an Ethernet-controlled FLIR A320 camera at 30 Hz.

Supplementary Text

Composition

When the NFC/PEDOT:PSS/Glycerol/DMSO emulsion is poured onto a Petri dish, we tracked the weight of the liquid emulsion during its drying. After all the water had evaporated during the drying, the weight continued to decrease as the DMSO and glycerol slowly evaporated. The weight of a sample, starting from solution , as a function of drying time can be seen in Fig. S1. After 200h the weight remains constant. The calculated weight ratios at this point are 19.2/7.3/73.5 for Celvios PH 1000, NFC and solvents (DMSO+glycerol) respectively.

Mechanical properties

Data from the tensile testing resulted in a Young's modulus of E = 0.68 ±0.06 GPa, a tensile strength of σ_T = 13 ±2 MPa, and strain at break of ε_T = 12.7 ±2.7 % for the composite. For comparison, a typical copy paper,[4] has a tensile modulus of 7 GPa, in the machine direction of the paper, which is about one order of magnitude higher than the composite. The tensile strength is about 4 times higher (50 MPa) and the strain at break is significantly smaller (1.35%). This means that the NFC-PEDOT composite film is weaker compared with a typical copy paper but this also demonstrates that the mechanical properties of the prepared film are indeed sufficient for a practical handling of the film.

From a structural perspective, it is also relevant to compare the composite film with unoriented films made of pure NFC and pure PEDOT:PSS. Henriksson et al. [5] used a filtering technique to prepare 60-80 µm thick NFC films with a modulus of E=13.2 GPa, a tensile strength of σ_T = 214 MPa and a strain at break of ε_T = 10.1%. For the PEDOT/PSS materials, Lang et al.[6] prepared 25 µm thick films by water casting. The so prepared films had a modulus of E=0.9 GPa and a tensile strength of σ_T = 22.2 MPa (at 55% RH and 23˚C). From these different results it is obvious that the mechanical properties of the prepared composite show a much closer resemblance to the PEDOT:PSS film than to the superior NFC films. This further supports one of the main findings in the present work, i.e. that the PEDOT:PSS is self-organizing as a shell around the NFC particles and, therefore, effectively prevents the formation of the strong NFC links in the film. The large amount of plasticizers (9.5% glycerol, 68.2% DMSO) in the composite may also significantly affect the mechanical properties, since they separate the polymers

and the reinforcing materials (PEDOT, PSS and NFC respectively) within the network and hence effectively reduces their interaction.

Electrical conductivity

The conductivity of the composite was measured as a function of temperature. Fig. S6 shows the data in an Arrhenius plot where the natural logarithm of the conductivity is plotted as a function of 1/T. From the slope of the obtained data it is possible to calculate the activation energy (ΔH) of the material. This is done by using the Arrhenius relationship described by equation S1. The activation energy of the 70 µm thick sample was calculated using the whole temperature interval and had a value of 3.75 meV. The activation energy of the 5 µm samples (before and after annealing) was calculated in the temperature range 0°C to -60°C where the slopes were linear. The activation energies were 1.2 meV and 2 meV, respectively.

$$\sigma(T) = \sigma_0 exp\left(\frac{-\Delta H}{kT}\right) \qquad \text{(S1)}$$

The 5 µm thick layer of NFC-PEDOT in Fig. S6 still contains a substantial amount of solvent (73.5wt% of DMSO+glycerol). In the high temperature region [212-277°K], the conductivity is 355 S/cm at room temperature and is proportional to 1/T. The charge transport is governed by nearest neighbor hopping mechanism (nn-H) with an activation energy of 1.2 meV.[7] At low temperatures [133-212°K], the conductivity deviates towards a variable range hopping (VRH) behaviour. Hence, the transport in the composite resembles that of the in-plane conductivity of a thin film (<1µm) of PEDOT:PSS on a flat substrate.[8]

Specific heat and thermal diffusivity

From the measured slopes of the thermocouple voltage (Fig. S7), the in-plane diffusivity $D = \pi f/(dlnV_{ac}/dx)^2 = (7.0 \pm 0.2) \times 10^{-3}$ cm^2/s . From the DSC value of the room temperature specific heat c = (1.32 ± 0.04) J/g·K (Fig. S7 inset) and density ρ = (1.26 ± 0.4) g/cm^3, we find that the in-plane thermal conductivity κ = Dcρ = (11.6 ± 1.0) mW/cm·K; the values of D, c, and κ are all very similar to what is reported for composites made of NFC and non-conducting polymer.[9]

Interplay between thermal and electrical properties

Fig. S8a is a picture taken with an infrared camera of a 160 µm thick NFC-PEDOT strip connected to two crocodile clips. When 2V electric potential difference is applied, a current of 1A passes in this organic resistor (current density is ~ 0.5 A/mm^2). The temperature reaches about 80°C in the resistor. Note the droplet of water deposited on the stripe shows a local lower temperature because of the endothermic evaporation process. Fig. S8b illustrates the kinetics of evaporation of the drop on the NFC-PEDOT resistor.

Thermoelectricity is anoother phenomenon that involves thermal and electrical transport. Thermoelectric properties of PEDOT:PSS have recently attracted the attention of the scientific community because of its potential for thermoelectric generators (TEG) due to its instrinsic low thermal conductivity.[10, 11] To date, the thermoelectric effect has only been demonstrated on thin films supported by a substrate. This architecture is not

desirable for a TEG since there is a large heat leakage through the substrate. Fig. S8c displays a infrared camera image of a 30 μm thick layer submitted to a temperature gradient obtained by two peltier devices in contact with both ends of the NFC-PEDOT free standing strip. The Seebeck coefficient of the organic composite material is ~17 μV/K, which is close to that of PEDOT:PSS reported elsewhere[12] and its thermoelectric figure of merit is 1.1×10^{-3} at room temperature. This NFC-PEDOT composite is the first free standing organic thermoelectrics that does not require a substrate. The latter typically leads to heat loss. Fig. S8d displays the electrical power generated versus temperature gradient.

Ionic conductivity

A purely ionic composite material with polystyrene sulphonic acid (PSSH) instead of PEDOT:PSS was fabricated in order to approximate the ionic conductivity of the NFC-PEDOT composite. This was done since the ionic conductivity is typically orders of magnitude lower than the electronic conductivity, making it difficult to decouple them.

Ionic conductivity is commonly measured using impedance spectroscopy by modeling the frequency response of the sample as a simple equivalent circuit containing a capacitor in series with a resistor. At high frequencies, the impedance of the capacitor will be negligible and the impedance will be comprised of the resistance only as indicated by a phase angle close to zero. The impedance at 1 kHz was used to calculate the ionic conductivity of the NFC-PSSH composite. Fig. S9a and S9b show the impedance and phase angle at various RH%. In order to confirm that the impedance consists solely of the ionic resistance at 1 kHz, the length of the NFC-PSSH samples were varied. Fig. S9c and S9d show the calculated ionic conductivity and phase angle for three samples with different lengths. Above 100 Hz, the values of conductivity are close to identical, indicating that the capacitance at the contacts has a small contribution to the impedance at these frequencies. Since the ionic conductivity of the composite films was measured in the lateral direction (which is not the standard procedure), a lateral liquid cell with similar thickness as the NFC-PSSH samples was manufactured. Fig. S10 shows the ionic conductivity of a 10 mM NaCl aqueous solution measured with a cylindrical (vertical) cell and the lateral cell. The values are within the same order of magnitude and differ by only a factor 1.8 at 1 kHz. This shows that the lateral measurement method gives a good approximation of the ionic conductivity.

Electrochemical characterization

The structures of the supercapacitor (or pseudocapacitor) cells are depicted in Fig. 4a. In order to determine the redox behavior of the NFC-PEDOT composite material, cyclic voltammetry was performed with an Ag/AgCl/KCl reference electrode. The resulting voltammogram (inset of Fig. 5c) show no distinct redox peaks in the potential range 0-0.6 V. The absence of redox peaks is characteristic for PEDOT:PSS and shows that no electrochemical reactions take place in the specified potential window. The square-like shape of the voltammogram indicates a highly capacitive behavior.

Chronoamperometric charge-discharge measurements were performed to investigate the switching behavior of the supercapacitor while applying a potential step. A physical

capacitor is usually modeled as an ideal capacitor in series with a resistance. The current behavior during charging of such an RC circuit is described by equation S2. Here, I_0 is the maximum current, V is the applied potential, R is the resistance, C is the capacitance and τ is the time constant.

$$I = I_0 e^{-t/\tau} = \frac{V}{R} e^{-t/RC} \qquad \text{(S2)}$$

Fig. S12 shows the charging of a 1 cm^2 supercapacitor as well as the simulated curve following equation S2. The measurement data and the simulation are in good agreement which corroborates with the results from the cyclic voltammetry. The simulation was performed by plugging the following values into equation S2: $R = 8.4\Omega$; $C = 39mF; \tau = 324ms$.

The chronoamperometric charge-discharge measurements were done over 500 cycles to determine the stability of the supercapacitors. Fig. 4d shows the charge during charging and discharging as well as the charge retention $\left(\frac{Q_{discharging}}{Q_{charging}} \times 100\right)$ for 11 such cycles.

Galvanostatic charge-discharge measurements were performed on 1 cm^2 supercapacitors with different thicknesses of the NFC-PEDOT films as well as on a pure Clevios PH 1000 film to determine their capacitance. The capacitance was calculated from the discharge-curve of each measurement using equation S3 where I is the current bias and $\frac{dV}{dt}$ is the slope of the discharge curve. For all measurements, the slope of both the charging and discharging curves were almost perfectly linear, making $\frac{dV}{dt}$ a constant. The specific capacitance can further be calculated by dividing the capacitance with the mass of the electroactive species of the material (equation S4).

$$C = \frac{I}{\frac{dV}{dt}} \qquad \text{(S3)}$$

$$C_p = \frac{C}{m} \qquad \text{(S4)}$$

In Fig. 4c the capacitance has been plotted as a function of the thickness of the NFC-PEDOT film for each supercapacitor.
The specific capacitance of the films was calculated to be 44 F/g when taking only the mass of PEDOT into account. The specific capacitance of pure Clevios PH 1000 was calculated to be 41 F/g. The agreement between the specific capacitance of the Clevios PH 1000 film and the NFC-PEDOT composites shows that there is no loss in performance of the material when blending the electroactive material with the non-conducting materials (NFC, glycerol and DMSO). The linear increase in capacitance with thickness shows that the specific capacitance of the NFC-PEDOT composite material is thickness-independent.

Galvanostatic charge-discharge measurements were also performed on a 71 cm^2 supercapacitor at different current bias (Fig. 4b) to show that it is possible to scale up the supercapacitors while maintaining high performance.

Electrochemical impedance spectroscopy (EIS) was performed on a 1 cm^2 supercapacitor in order to evaluate its frequency dependence. The impedance of an electrochemical system is often modeled as an equivalent circuit containing ideal circuit elements in order to decouple different parameters. In such an equivalent circuit, each circuit element corresponds to a physical process taking place in the system while

applying an AC voltage. We modeled our system using the Cole-Cole equivalent circuit with an additional resistor. Fig. S13 shows the Nyquist plot of the impedance of the supercapacitor as well as a simulation made from a modified Cole-Cole circuit (also shown in Fig. S13). The impedance of the constant phase element (CPE) has the form $Z_{CPE} = 1/[A(j\omega)^{\alpha}]$ where $0 < \alpha < 1$. The CPE is associated with the transport and storage of ions in the NFC-PEDOT films. The parameters of the different circuit elements from the simulation are shown in table S3.

The fact that the parameter α is smaller than 0.5 indicates that the CPE is mostly resistive and can thus be approximated as a resistor with the resistance 1/A. By adding this resistance to the uncompensated electrolyte resistance we arrive at the same resistance as was measured in the amperometric charging measurements. Furthermore, the capacitor C_2 has the same value as the capacitance measured in the charging measurements. This leads us to believe that C_2 represents the capacitance of the composite and the CPE represents resistance of the charge transport and transfer in the composite. From this model the minimum theoretical time constant can be calculated as: $\tau = R \times C = 1/A \times C_2 = 40ms$.

Since the parameter α is not zero, there is a frequency dependence on the charge transfer resistance. This is very plausible since the electrical conductivity of the composite changes as the redox reaction takes place during charging of the capacitor. When plotting the real part impedance of the CPE vs. frequency, it can be seen that the impedance is almost constant at high frequencies but increases quickly for f<10 Hz. These frequencies correspond to the measured time constant of the capacitor.

We did not attempt to give physical interpretations to the circuit elements R_1 and C_1. These could be the result of double layer formation on the gold surfaces as well as other parasitic capacitances.

Water stability

The NFC-PEDOT composite shows superior water stability over pure PEDOT:PSS (Clevios PH 1000). Fig. S14 shows a dried film of PEDOT:PSS and the NFC-PEDOT composite being submerged in DI-water. The composite immediately soaks up the water and sinks while the PEDOT:PSS film is significantly more hydrophobic and, thus, floats (Fig. S14b). The PEDOT:PSS film slowly swells and sinks (Fig. S14c). After 10 minutes, the PEDOT:PSS film has swollen to several times its original volume (Fig. S14d). After 30 minutes the PEDOT:PSS film start to disintegrate (Fig. S14e). The composite film on the other hand can sustain immersion in water for several months without any visible degradation.

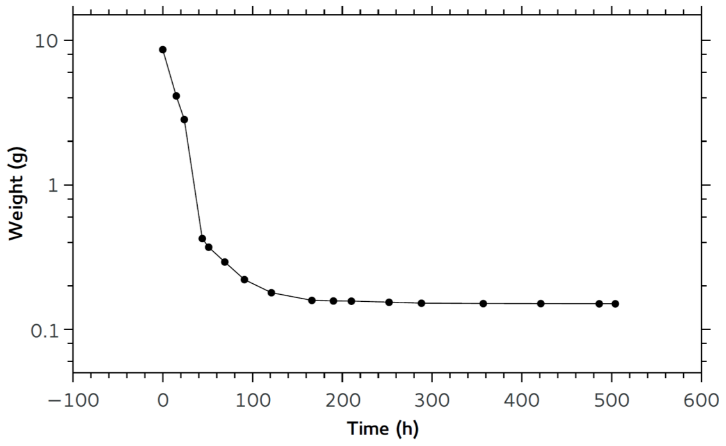

Fig. S1

Evolution of the weight of the sample versus time during the drying step.

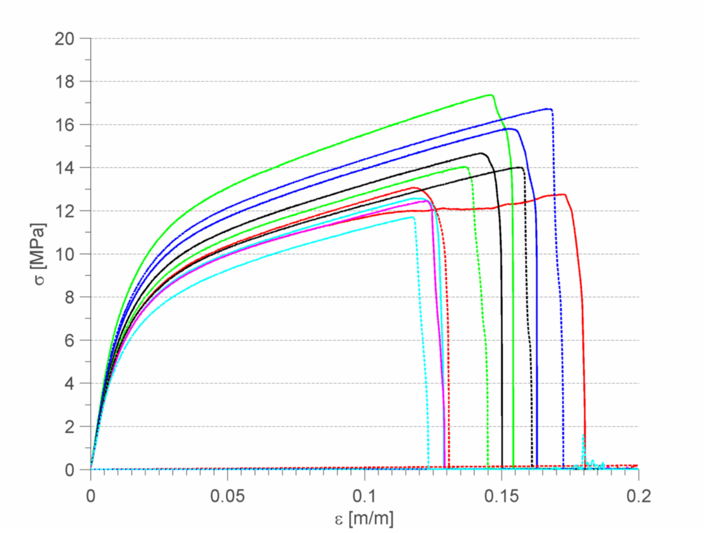

Fig. S2

Tensile strength vs strain for the NFC-PEDOT samples

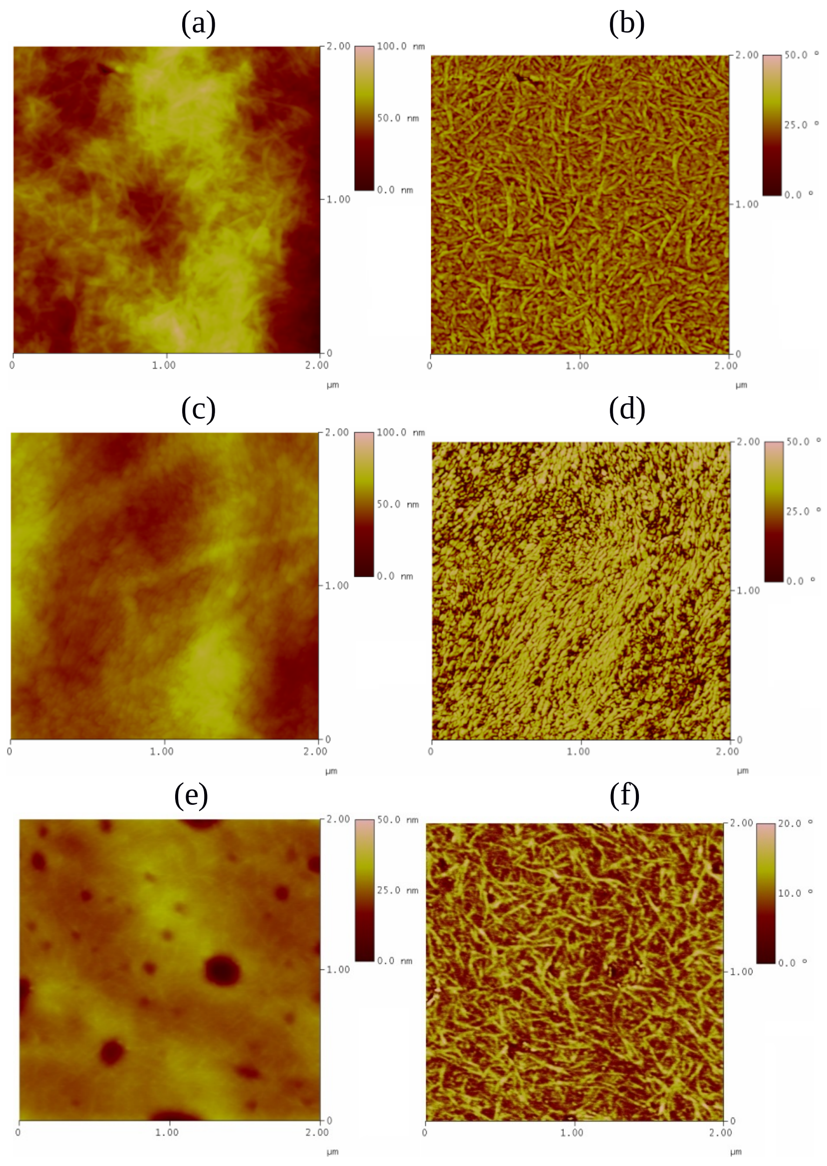

Fig. S3

(a) Topography and (b) phase AFM image of a ~70 µm thick film of vacuum-dried NFC-PEDOT composite. (c) topography and (d) phase AFM image of a ~10-20 µm thick film of NFC made from an aqueous suspension dried in ambient atmosphere. (e) topography and (f) phase AFM image of a thick film of NFC-PSSH made from an aqueous suspension and then vacuum-dried. The dimension of the images is 2×2µm.

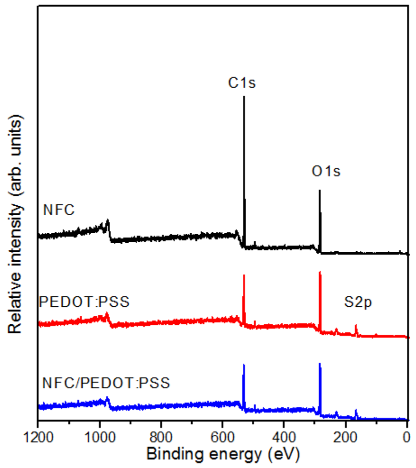

Fig. S4

XPS (Al Kα) wide scan for three samples: NFC deposited on Au substrate, free standing film of PEDOT:PSS; free standing film of NFC/PEDOT:PSS.

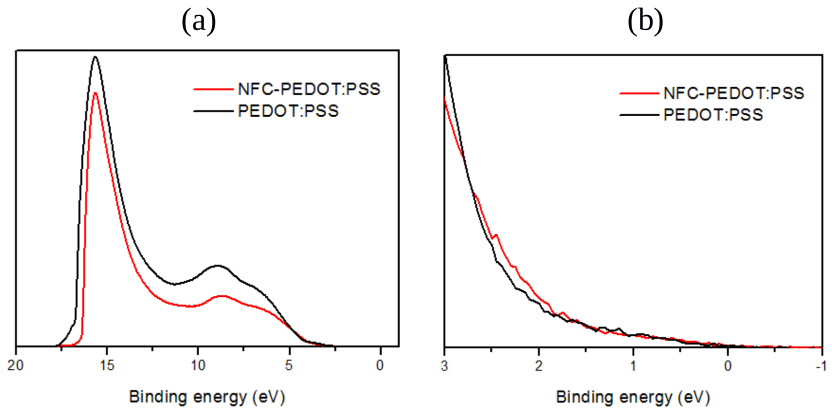

Fig. S5

(a) UPS (HeI) wide scan spectra of the NFC-PEDOT and PEDOT:PSS films; and (b) a zoom close to the Fermi level of the spectrometer (binding energy of 0 eV).

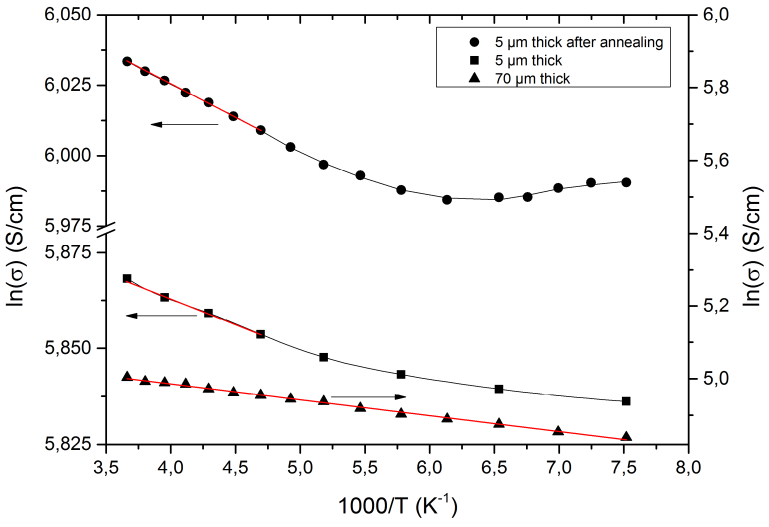

Fig. S6

Log of the electrical conductivity versus 1000/T for a 5µm-thick layer possesses a complex non-linear behavior (squares). After vacuum annealing (circles), the conductivity is characteristic of a metallic-type transport at low temperature. For 70 µm-thick layer, the evolution is linear (triangles) and indicates a nn-H mechanism.

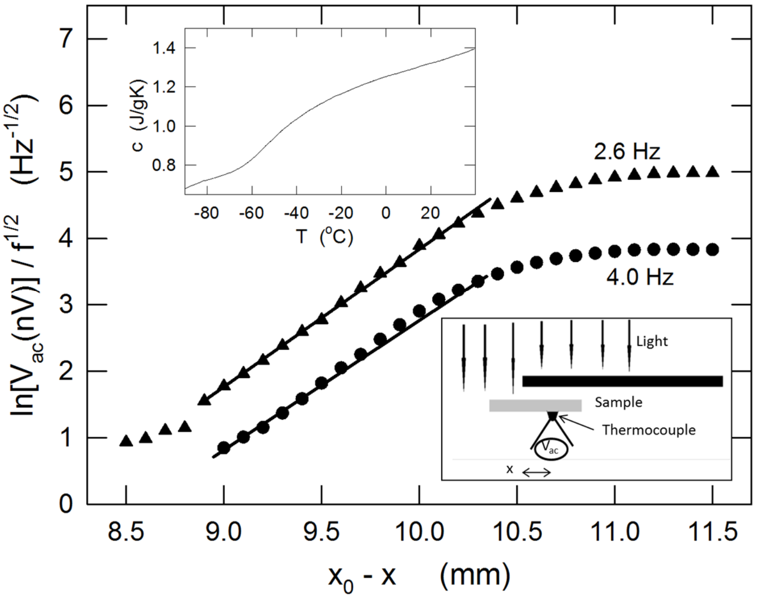

Fig. S7

Position dependence of the oscillating thermocouple signal (V_{ac}) for two different chopping frequencies for a 30 μm thick sample; x = distance between the edge of the screen and the thermocouple, and x_0 = constant offset. Top inset: specific heat of a 14 mg pellet measured with differential scanning calorimetry at a scanning rate of 18 K/minute. Bottom inset: schematic of the experimental layout.

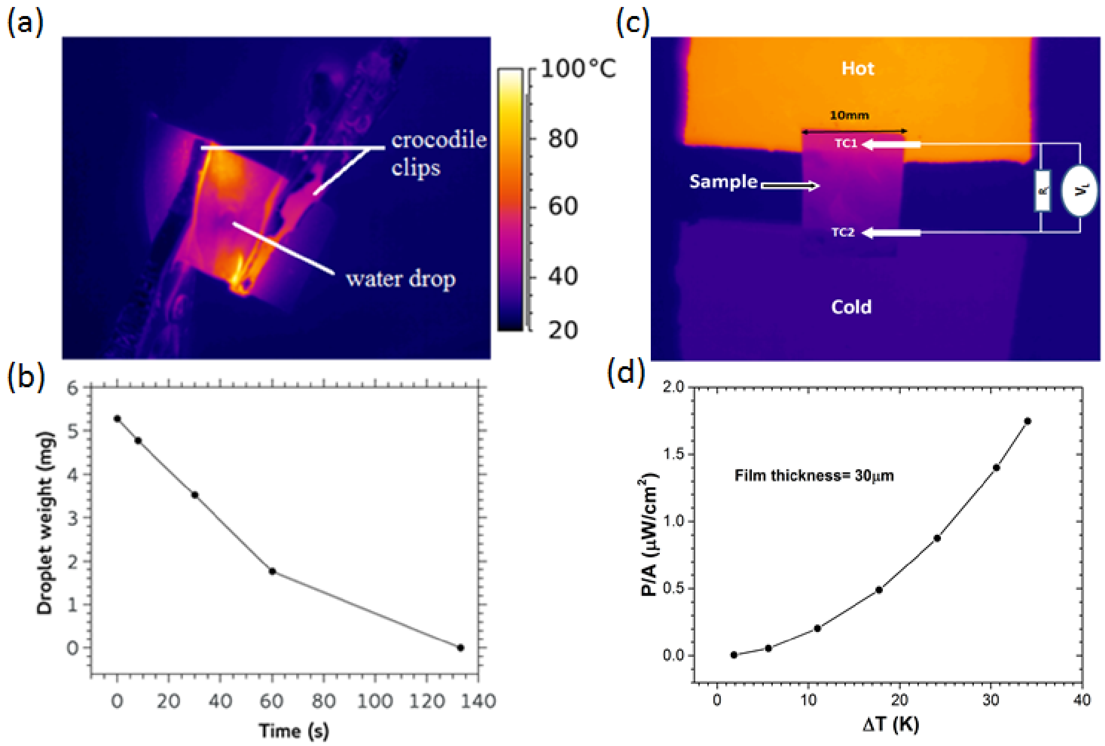

Fig. S8

(a) Infrared camera image of a 160 µm thick sample connected to two crocodile clips and subjected to a current of 1 A. A drop of water is deposited in the middle. (b) Evolution of the weight of the drop versus time showing the evaporation of the drop. (c) Infrared camera image of a free standing 30 µm thick sample connected thermally to a hot and a cold peltier device. The temperature is read with thermocouples (TC1 and TC2). (d) The generated electrical power versus temperature gradient is a result of a thermoelectric effect. Maximum power transfer occurred at a load (R_L) of 10 Ω.

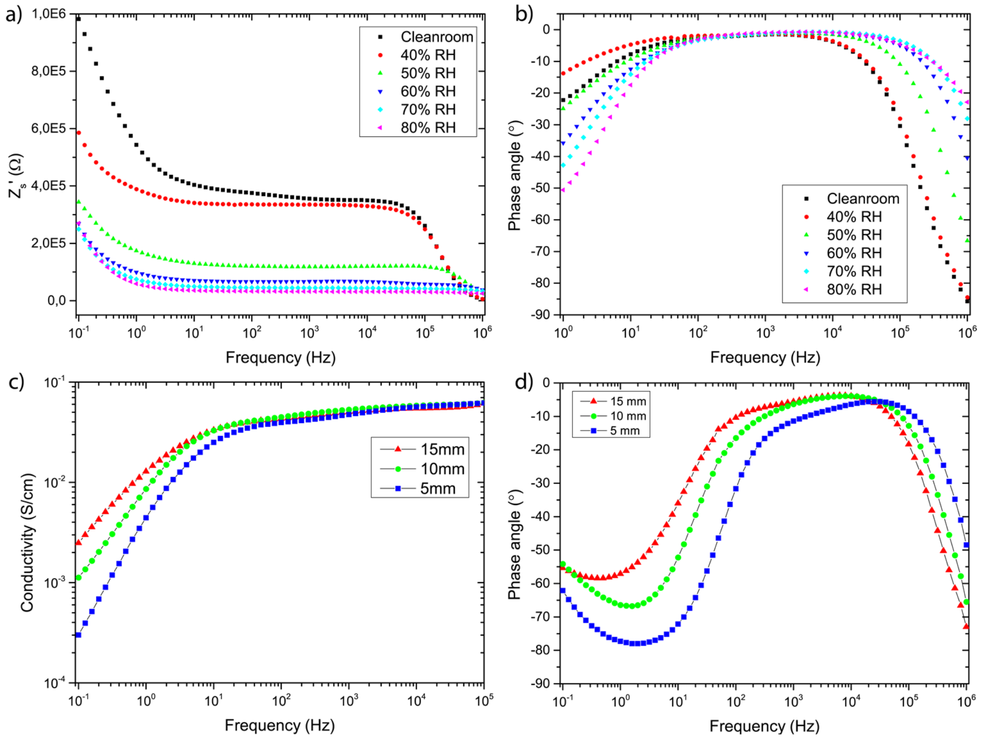

Fig. S9

Real part impedance (a) and phase angle (b) of NFC-PSSH measured at different levels of relative humidity. Ionic conductivity (c) and phase angle (d) of NFC-PSSH with different sample length.

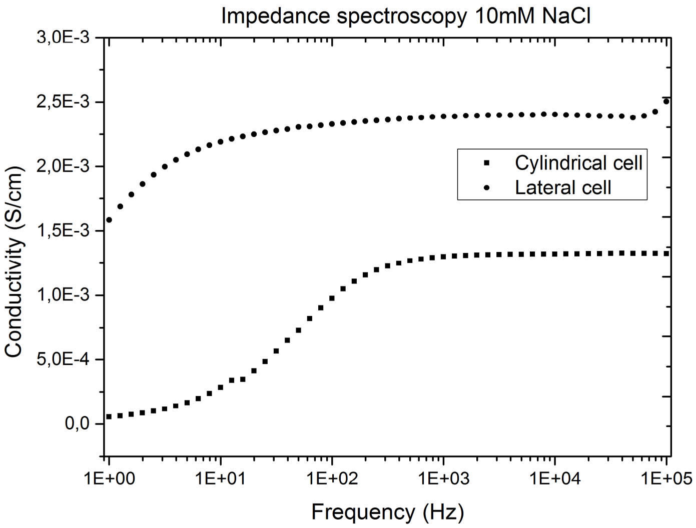

Fig. S10

Ionic conductivity of 10 mM NaCl water solution measured with impedance spectroscopy using a lateral cell and vertical cell.

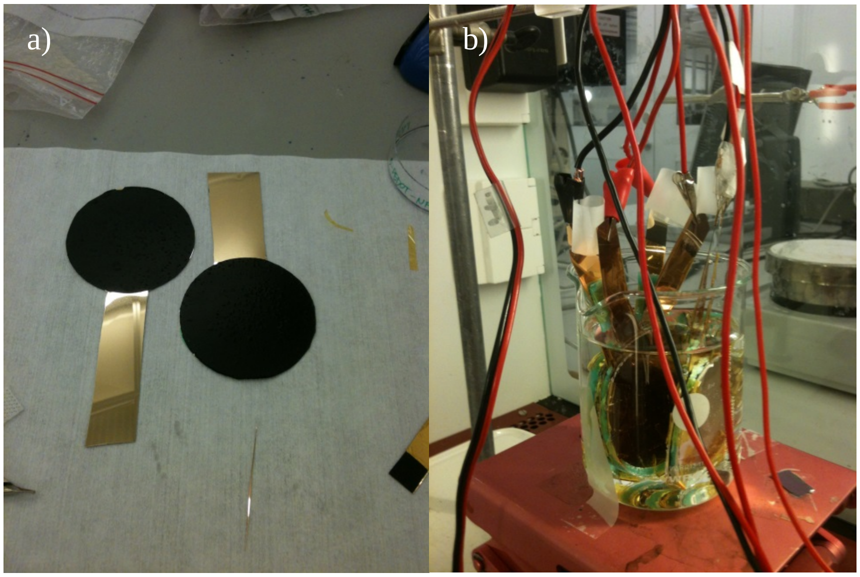

Fig. S11

2x24 cm^2 NFC-PEDOT films laminated on Au on PET (a) and a photograph of the supra-farad supercapacitor device (b).

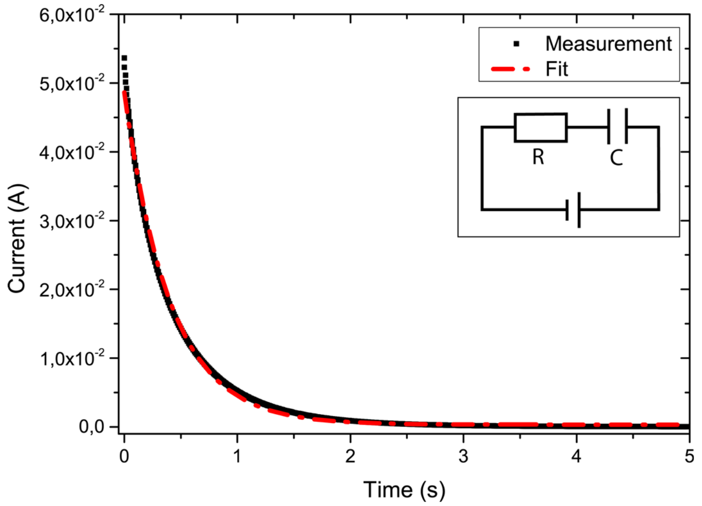

Fig. S12

Charging-curve of a NFC-PEDOT supercapacitor and a simulated and fitted curve based on the equivalent circuit in the inset.

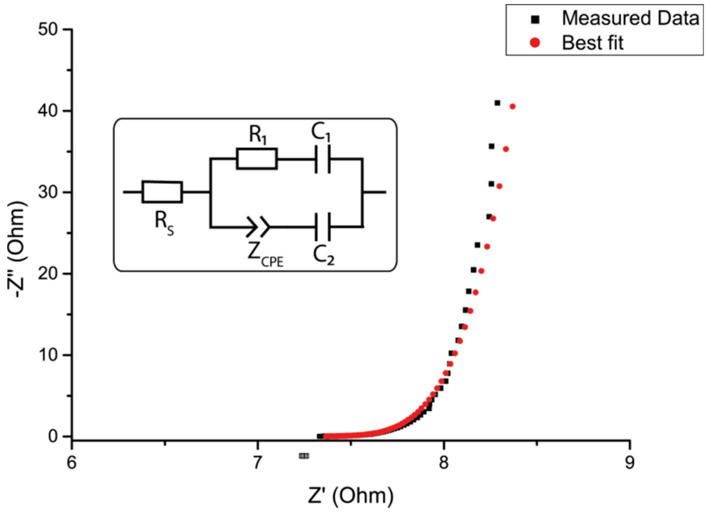

Fig. S13

The Nyquist plot of a NFC-PEDOT supercapacitor and a simulated and fitted curve based on the equivalent circuit in the inset.

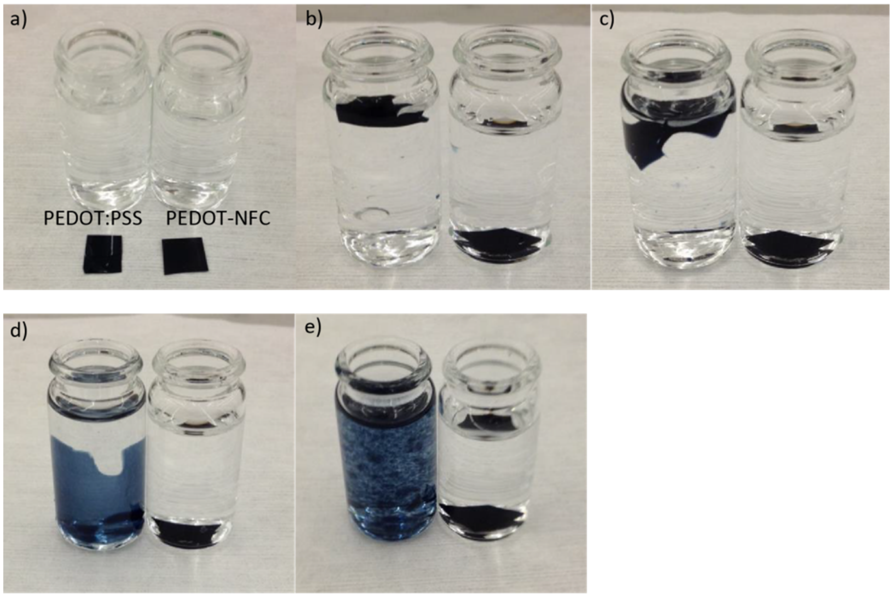

Fig. S14

Behavior of the dry PEDOT:PSS and the NFC-PEDOT nanopaper when put in water.

Table S1.

Mechanical measurements for different samples.

sample	Grammage [g/m²]	Thickness [µm]	Density [kg/m³]	Stress at Break in Tension [MPa]	Elastic Modulus [GPa]	Tensile Energy Absorption per Unit Area [kJ/m²]	Strain at Break [%]
(1)	147,4	120,6	1222	12,77	0,62	0,226	17,3
(2)	147,4	120,6	1222	17,39	0,85	0,251	14,6
(3)	147,4	120,6	1222	15,83	0,74	0,242	15,1
(4)	147,4	120,6	1222	14,69	0,70	0,207	14,2
(5)	147,4	120,6	1222	12,60	0,64	0,152	11,9
(6)	147,4	120,6	1222	12,48	0,63	0,150	12,2
(7)	147,4	120,6	1222	13,09	0,66	0,158	11,7
(8)	147,4	120,6	1222	14,06	0,66	0,188	13,7
(9)	147,4	120,6	1222	16,75	0,76	0,267	16,6
(10)	147,4	120,6	1222	14,02	0,64	0,209	15,7
(11)	147,4	120,6	1222	11,71	0,62	0,133	11,7
average	**147,4**	**120,6**	**1222**	**13,53**	**0,66**	**0,180**	**13,4**
STDAV		5,4		1,65	0,05	0,046	2,0

Table S2.

Parameters of the regression analysis of the conductivity vs. thickness measurement shown in Fig. 4b.

Equation	y = a + b*x
Weight	No Weighting
Residual Sum of Squares	2093,265
Pearson's r	0,06666
Adj. R-Square	-0,08606

	Value	Standard Error
Intercept	135,4797	9,70873
Slope	0,01664	0,07508

Table S3.

Parameters of the model of the electrochemical system in Fig. S13.

R_s = 7.3Ω	A = 0.96	α = 0.26	C_2 = 39mF	R_1 = 323.7$m\Omega$	C_1 = 827µF

References:

[1] Y. Wang, M. Chung, T. N. O'Neal, J. W. Brill, Synthetic Metals 1992, 46, 307.
[2] I. Hatta, Y. Sasuga, R. Kato, A. Maesono, Review of Scientific Instruments 1985, 56, 1643.
[3] H. Zhang, Y. Yao, M. M. Payne, J. E. Anthony, J. W. Brill, Applied Physics Letters 2014, 105, 073302.
[4] J. Alfthan, Nordic Pulp and Paper Research Journal 2010, Volume 25 351.
[5] M. Henriksson, L. A. Berglund, P. Isaksson, T. Lindström, T. Nishino, Biomacromolecules 2008, 9, 1579.
[6] U. Lang, N. Naujoks, J. Dual, Synthetic Metals 2009, 159, 473.
[7] A. M. Nardes, M. Kemerink, R. A. J. Janssen, Phys Rev B 2007, 76, 085208.
[8] A. M. Nardes, R. A. J. Janssen, M. Kemerink, Advanced Functional Materials 2008, 18, 865.
[9] Y. Shimazaki, Y. Miyazaki, Y. Takezawa, M. Nogi, K. Abe, S. Ifuku, H. Yano, Biomacromolecules 2007, 8, 2976.
[10] O. Bubnova, Z. U. Khan, A. Malti, S. Braun, M. Fahlman, M. Berggren, X. Crispin, Nat Mater 2011, 10, 429.
[11] G. H. Kim, L. Shao, K. Zhang, K. P. Pipe, Nat Mater 2013, 12, 719.
[12] S. v. Reenen, M. Kemerink, Organic Electronics 2014, 15, 2250.
